# Stromal p16 expression is significantly increased in malignant ovarian neoplasms

**DOI:** 10.18632/oncotarget.11660

**Published:** 2016-08-27

**Authors:** Nara Yoon, Gun Yoon, Cheol Keun Park, Hyun-Soo Kim

**Affiliations:** ^1^ Department of Pathology, The Catholic University of Korea Incheon St. Mary's Hospital, Incheon, Republic of Korea; ^2^ Shinsegae Women's Hospital, Daegu, Republic of Korea; ^3^ Department of Pathology, Severance Hospital, Yonsei University College of Medicine, Seoul, Republic of Korea

**Keywords:** p16, ovary, neoplasm, peritumoral stroma, immunohistochemistry, Pathology Section

## Abstract

Alterations in p16 protein expression have been reported to be associated with tumor development and progression. However, p16 expression status in the peritumoral stroma has been rarely investigated. We investigated the stromal p16 expression in ovarian neoplasms using immunohistochemistry, and differences in the expression status depending on the degree of malignancy and histological type were analyzed. This study included 24, 21, and 46 cases of benign, borderline, and malignant ovarian lesions, respectively, of which 29, 25, and 32 cases were serous, mucinous, and endometriosis-associated lesions. Most benign lesions showed negative or weak expression, whereas borderline lesions showed focal, moderate expression. Malignant lesions showed markedly elevated stromal p16 expression compared with benign or borderline lesions. There were significant differences in stromal p16 expression between benign and borderline lesions (*P* < 0.001) and between borderline and malignant lesions (*P* < 0.001). These significances remained when analysis was performed based on lesion classification as serous, mucinous, and endometriosis-associated. In contrast, differences in stromal p16 expression among the histological types were not significant. Stromal p16 expression in ovarian neoplasms was absent or weak in benign and focal, moderate in borderline lesions, whereas malignant lesions exhibited diffuse, moderate-to-strong p16 immunoreactivity. Our observations suggest that stromal p16 expression is involved in the development of ovarian carcinoma. Further studies are necessary to confirm our preliminary results.

## INTRODUCTION

p16 is the principal member of the INK4 family of cyclin-dependent kinase (CDK) inhibitors [1]. As a regulatory protein of the cell cycle, p16 is involved in the G1-to-S phase transition. Upon binding to CDK4/6, p16 inhibits formation of the cyclin D1-CDK4/6 complex and CDK4/6-mediated phosphorylation of the retinoblastoma (RB) protein. Once RB is phosphorylated, the E2F-RB complex dissociates, leading to reduced growth-suppressor activity of RB [2]. Like RB, p16 is known as a tumor suppressor. p16 maintains RB family members in a hypophosphorylated state [2-5]. However, it is difficult to explain many aspects of p16 function and regulation by its well-known function as a tumor suppressor alone. In addition, molecular pathways responsible for p16 function and expression have not yet been determined.

Conflicting patterns of p16 expression have been reported, which further complicates the understanding of its biological and pathological roles. In different types of neoplasms, p16 expression is either lost or downregulated [6-9], or clearly overexpressed [10-13]. p16 expression has been analyzed in some studies of gynecological malignancy. According to the 2014 World Health Organization (WHO) Blue Book, 60% of ovarian high-grade serous carcinomas show diffuse, strong p16 expression [14]. p16 and p53 expression levels are used as differential markers to distinguish high-grade serous carcinoma from other histological types of ovarian carcinoma. It is well known that p16 overexpression occurs in human papillomavirus (HPV)-related tumors [1, 14]. p16 overexpression indicates high-risk HPV infection, not only in uterine cervical carcinoma and head and neck carcinoma, but also in high-grade squamous intraepithelial lesions (HSILs) of the vulvovaginal and anogenital regions; therefore, p16 is used as a diagnostic marker for both HSIL and invasive squamous cell carcinoma [1, 15]. Similarly, in breast carcinoma, high p16 immunoreactivity is significantly correlated with more undifferentiated and malignant phenotypes (i.e., estrogen receptor negativity and higher nuclear grade) [13]. Moreover, p16 staining intensity and expression are significantly higher in uterine leiomyosarcoma compared with benign uterine leiomyoma or uterine smooth muscle tumors of uncertain malignant potential [16].

Recently, during the routine diagnosis of surgically resected ovarian neoplasms, we noticed p16 expression in the peritumoral stroma. The levels of p16 expression in the stromal cells varied depending on degree of malignancy and histological type. Although p16 is commonly used as a biomarker for diagnosing gynecological malignancies, its expression in the stromal component of ovarian neoplasms has never been studied. In this study, we examined stromal p16 expression in benign, borderline, and malignant ovarian neoplasms by immunohistochemistry to determine whether a significant difference exists in stromal p16 immunoreactivity according to degree of malignancy and/or histological type.

## RESULTS

### Patient demographics

This preliminary study was conducted with 91 patients who underwent surgical excision for benign, borderline, or malignant neoplasms of the ovary from March 2015 to May 2016. The age of patients ranged from 23 to 61 years (median, 42 years) in patients with benign lesions, from 25 to 66 years (median, 48 years) in patients with borderline lesions, and from 28 to 82 years (median, 53 years) in patients with malignant lesions. None of the patients received pre-operative neoadjuvant chemotherapy, radiation therapy, or concurrent chemoradiation therapy. Classification of all 91 cases according to degree of malignancy of ovarian neoplasms resulted in 24 (26.4%) cases in the benign group, 21 (23.1%) cases in the borderline group, and 46 (50.5%) cases in the malignant group. According to histological type, the 91 cases were classified into serous type (29 cases; 31.9%), mucinous type (25 cases; 27.5%), Brenner type (5 cases; 5.5%), and endometrioid-associated type (32 cases; 35.2%). The endometriosis-associated type included the endometrioid type (17 cases; 53.1%), clear cell type (8 cases; 25.0%), and seromucinous type (7 cases; 21.9%). Seromucinous tumors are defined as benign cystic neoplasm (seromucinous cystadenoma), non-invasive, proliferative epithelial neoplasm (seromucinous borderline tumor, formerly known as endocervical-type or Mullerian mucinous borderline tumor) or carcinoma (seromucinous carcinoma) with two or more epithelial cell types, all accounting for at least 10% of the epithelium. The epithelium lining the cysts or papillae is composed mostly of serous or endocervical-type mucinous epithelium, but endometrioid, squamous, and clear cells may be seen [14]. Histological types were classified following the criteria of the WHO Classification of Tumours of Female Reproductive Organs, revised in 2014 [14].

### Stromal p16 expression in benign, borderline, and malignant ovarian neoplasms

**Figure 1:**
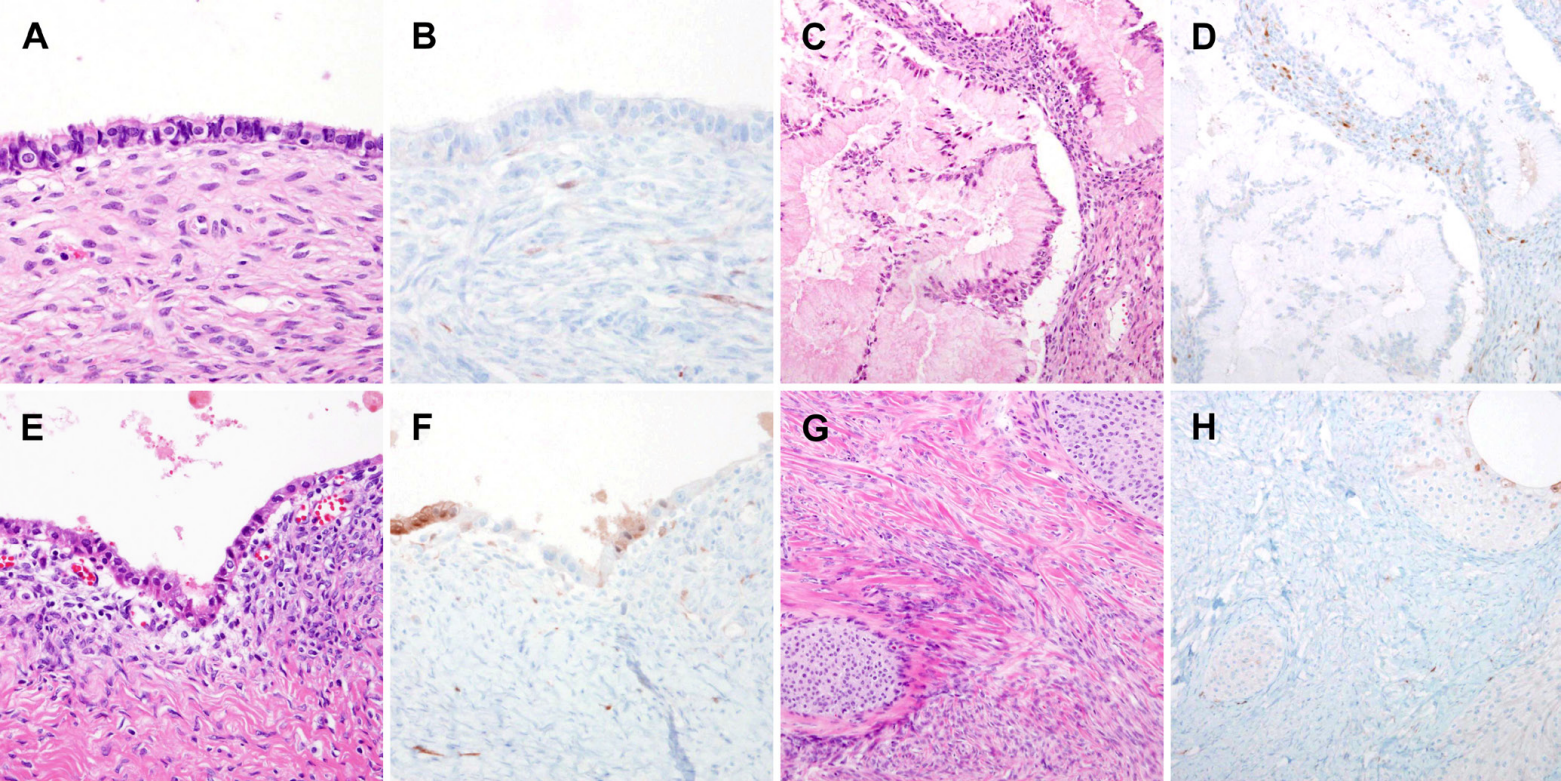
Stromal p16 overexpression in benign ovarian tumors **A.** Serous cystadenoma. **B.** A few stromal cells displayed faint p16 immunoreactivity. **C.** Mucinous cystadenoma. **D.** Some scattered inflammatory cells were positive for p16. **E.** Endometriotic cyst. **F.** Some endometrial-type epithelial cells exhibited weak cytoplasmic p16 immunoreactivity. **G.** Benign Brenner tumor. **H.** Evident lack of stromal p16 expression.

p16 immunostaining scores of benign, borderline, and malignant ovarian neoplasms are presented in Table 1. Representative photomicrographs of stromal p16 expression in benign ovarian neoplasms are presented in Figure 1. All 24 cases of benign ovarian neoplasm showed a p16 immunostaining score of 3 or less. Of the 24 cases of benign lesions, 14 (58.3%) cases showed no p16 expression, whereas 7 (29.2%) cases, 2 (8.3%) cases, and 1 (4.2%) case had scores of 1, 2, and 3, respectively. No significant difference was observed in stromal p16 expression status among the different histological types (*P* = 0.688). Of 6 cases of endometriotic cysts, 4 (66.7%) were found to have patchy and weak cytoplasmic p16 immunoreactivity in endometrial-type epithelial cells lining the cystic space. Similarly, 1 of 3 (33.3%) benign Brenner tumors showed patchy and weak p16 expression in the tumor cell cytoplasm.

**Table 1:** Stromal p16 expression in benign, borderline, and malignant ovarian lesions

Category	Pathological diagnosis	Total	p16 immunostaining score							*P* value
			0	1	2	3	4	6	9	
Benign	Serous cystadenoma/adenofibroma	6	4 (66.6)	1 (16.7)	1 (16.7)	0 (0.0)	0 (0.0)	0 (0.0)	0 (0.0)	0.688
	Mucinous cystadenoma/adenofibroma	7	4 (57.1)	2 (28.6)	0 (0.0)	1 (14.3)	0 (0.0)	0 (0.0)	0 (0.0)	
	Endometriosis/endometrioid cystadenoma	6	2 (33.3)	3 (50.0)	1 (16.7)	0 (0.0)	0 (0.0)	0 (0.0)	0 (0.0)	
	Benign Brenner tumor	3	3(100.0)	0 (0.0)	0 (0.0)	0 (0.0)	0 (0.0)	0 (0.0)	0 (0.0)	
	Seromucinous cystadenoma	2	1 (50.0)	1 (50.0)	0 (0.0)	0 (0.0)	0 (0.0)	0 (0.0)	0 (0.0)	
Borderline	Serous borderline tumor	7	0 (0.0)	1 (14.3)	2 (28.6)	2 (28.6)	2 (28.6)	0 (0.0)	0 (0.0)	0.662
	Mucinous borderline tumor	8	0 (0.0)	1 (12.5)	2 (25.0)	3 (37.5)	2 (25.0)	0 (0.0)	0 (0.0)	
	Endometrioid borderline tumor	2	0 (0.0)	0 (0.0)	1 (50.0)	1 (50.0)	0 (0.0)	0 (0.0)	0 (0.0)	
	Borderline Brenner tumor	1	0 (0.0)	0 (0.0)	1 (100.0)	0 (0.0)	0 (0.0)	0 (0.0)	0 (0.0)	
	Seromucinous borderline tumor	2	0 (0.0)	0 (0.0)	0 (0.0)	2 (100.0)	0 (0.0)	0 (0.0)	0 (0.0)	
	Clear cell borderline tumor	1	0 (0.0)	0 (0.0)	1 (100.0)	0 (0.0)	0 (0.0)	0 (0.0)	0 (0.0)	
Malignant	High-grade serous carcinoma	12	0 (0.0)	0 (0.0)	0 (0.0)	0 (0.0)	1 (8.3)	7 (58.4)	4 (33.3)	0.648
	Low-grade serous carcinoma	4	0 (0.0)	0 (0.0)	0 (0.0)	0 (0.0)	1 (25.0)	3 (75.0)	0 (0.0)	
	Mucinous carcinoma	10	0 (0.0)	0 (0.0)	0 (0.0)	0 (0.0)	0 (0.0)	4 (40.0)	6 (60.0)	
	Endometrioid carcinoma	9	0 (0.0)	0 (0.0)	0 (0.0)	0 (0.0)	0 (0.0)	1 (11.1)	8 (88.9)	
	Malignant Brenner tumor	1	0(0.0)	0 (0.0)	0 (0.0)	0 (0.0)	0 (0.0)	0 (0.0)	1 (100.0)	
	Seromucinous carcinoma	3	0(0.0)	0 (0.0)	0 (0.0)	0 (0.0)	0 (0.0)	2 (66.7)	1 (33.3)	
	Clear cell carcinoma	7	0(0.0)	0 (0.0)	0 (0.0)	0 (0.0)	1 (14.3)	4 (57.1)	2 (28.6)	

**Figure 2:**
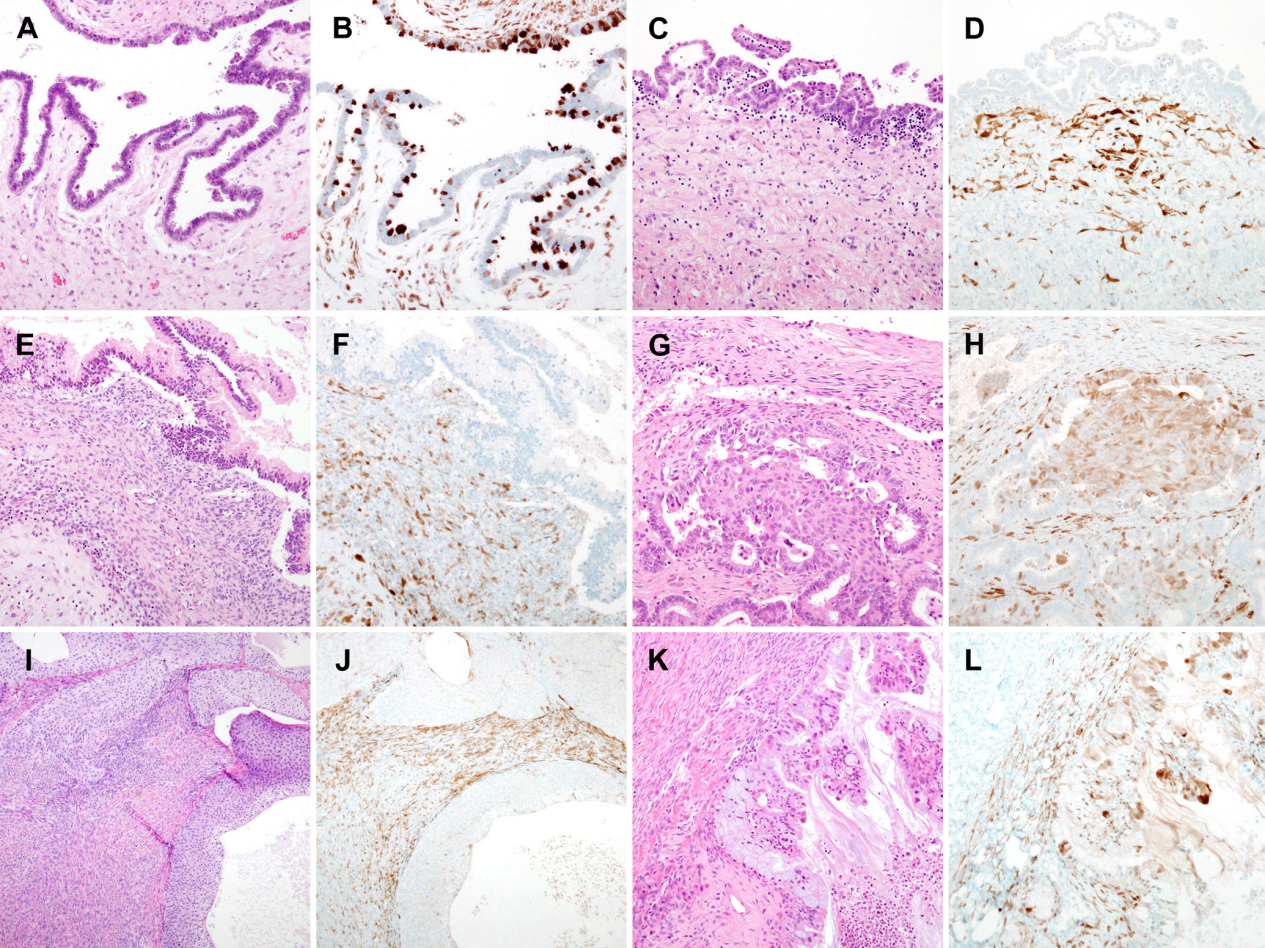
Stromal p16 overexpression in borderline ovarian tumors **A.** Serous borderline tumor. **B.** Tumor cells displaying patchy but strong cytoplasmic p16 immunoreactivity. p16 expression was weaker in stromal cells than in tumor cells. **C.** Serous borderline tumor, another case. **D.** In this case, the tumor cells did not react with a p16 antibody. Spindle- or stellate-shaped stromal cells exhibited weak-to-moderate p16 immunoreactivity. **E.** Mucinous borderline tumor. **F.** Similar to the serous borderline tumors, most stromal cells showed weak cytoplasmic p16 immunoreactivity. **G.** Endometrioid borderline tumor. **H.** Some tumor cells, as well as the central squamous morule, displayed weak cytoplasmic p16 immunoreactivity. Focal, moderate p16 expression was observed in the stroma. **I.** Borderline Brenner tumor. **J.** Lack of p16 expression in tumor cells. Stromal cells showed patchy, moderate p16 immunoreactivity. **K.** Seromucinous borderline tumor. **L.** A few tumor cells showed strong nuclear p16 immunoreactivity. The stromal cells displayed weak cytoplasmic p16 immunoreactivity.

Representative photomicrographs of stromal p16 expression in borderline ovarian neoplasms are presented in Figure 2. Of the 21 cases of borderline ovarian neoplasms, 12 (57.1%) showed p16 immunostaining scores of 3 or greater. Two (28.6%) cases of serous borderline tumor and 2 (25.0%) mucinous borderline tumors had p16 immunostaining scores of 4. While p16 immunostaining scores of serous and mucinous types varied from 1 to 4, those of the other 4 histological types were 2 or 3. These results might reflect the smaller number of cases with these histological types compared with the serous or mucinous types. Consistent with the benign lesions, no significant difference was observed in stromal p16 expression status among the different histological types of borderline ovarian neoplasms (*P* = 0.662). Even though 2 (28.6%) serous borderline tumors, 1 (50.0%) endometrioid borderline tumor, and 1 (50.0%) seromucinous borderline tumor exhibited moderate-to-strong p16 staining intensity in the tumor cells, none of the cases showed diffuse expression.

**Figure 3:**
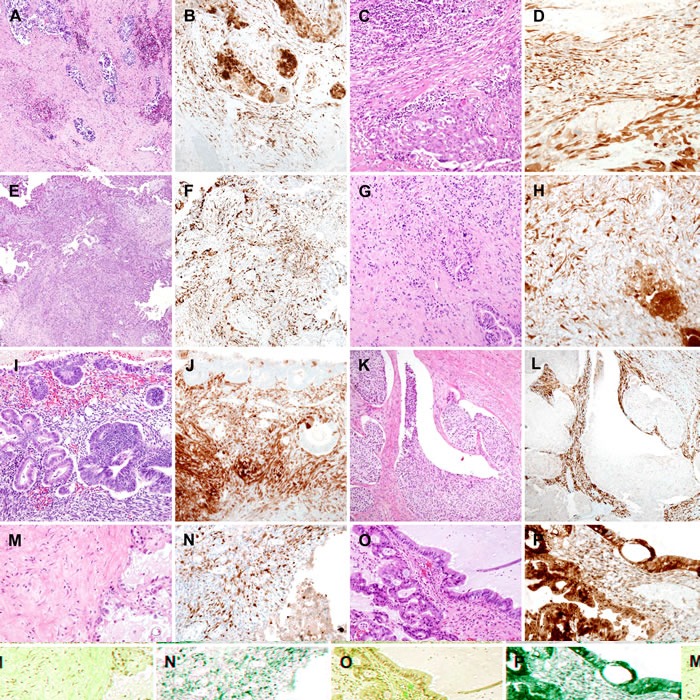
Stromal p16 overexpression in malignant ovarian tumors **A.**-**D.** High-grade serous carcinoma. **B.** The tumor cells demonstrated strong nuclear and cytoplasmic p16 expression. The spindle-shaped stromal cells displayed the same degree of p16 staining intensity as the tumor cells. **C.** High-grade serous carcinoma. **D.** In another high-grade serous carcinoma case, the stromal cells exhibited moderate-to-strong p16 expression, both in their nuclei and cytoplasm. **E.** Low-grade serous carcinoma. **F.** p16 immunostaining highlighted the stromal cells, which expressed p16 uniformly. **G.** Mucinous carcinoma with a destructive stromal-invasive pattern. **H.** Both tumor cells (lower right corner) and stromal cells (upper left corner) strongly expressed p16. **I.** Endometrioid carcinoma. **J.** The hypercellular stroma showed diffuse, intense p16 immunoreactivity. **K.** Malignant Brenner tumor. **L.** p16 immunostaining highlighted the stroma, which separated into irregular-shaped tumor cell nests and sheets. **M.** Clear cell carcinoma. **N.** The tumor cells (lower right corner) displayed weak cytoplasmic p16 expression, whereas the stromal cells (left half) showed moderate-to-strong nuclear p16 immunoreactivity. **O.** Seromucinous carcinoma. **P.** Similar to high-grade serous carcinoma, the tumor cells strongly reacted with p16. The stromal cells showed diffuse, moderate p16 staining.

Representative photomicrographs of stromal p16 expression in malignant ovarian neoplasms are presented in Figure 3. Of the 24 cases of benign ovarian neoplasms and the 21 cases of borderline ovarian neoplasms, only 4 (8.9%) borderline lesions showed p16 immunostaining scores of 4 or more, whereas all 46 (100.0%) cases of malignant ovarian neoplasms showed p16 immunostaining scores of 4 or greater. Moreover, 43 (93.5%) cases showed p16 immunostaining scores of 6 or greater. For 6 (60.0%) cases of mucinous carcinoma and 8 (88.9%) cases of endometrioid carcinoma, p16 expression in the stroma was diffuse and strong, with an immunostaining score of 9.

### Differences in stromal p16 expression according to degree of malignancy of ovarian neoplasms

The mean p16 immunostaining scores of benign, borderline, and malignant ovarian neoplasms were 0.6, 2.7 and 7.3, respectively. To analyze differences in stromal p16 expression between groups classified by degree of malignancy, a linear-by-linear association test was performed (Table 2). A significant difference was observed in stromal p16 expression between the benign and borderline groups (*P* < 0.001). Moreover, stromal p16 expression differed significantly between the borderline and malignant groups (*P* < 0.001). The linear-by-linear association test performed according to the histological type of each group also revealed statistical significances between the benign and borderline (*P* = 0.008) and the borderline and malignant (*P* < 0.001) serous neoplasms (Table 3). For the mucinous type, a significant difference was also noted in stromal p16 expression when comparing the benign and borderline groups with the borderline and malignant mucinous neoplasms (*P* = 0.008 for benign *versus* borderline and *P* < 0.001 for borderline *versus* malignant). In addition, endometriosis-associated types showed consistent stromal p16 expression patterns according to degree of malignancy (*P* = 0.004 for benign *versus* borderline; *P* < 0.001 for borderline *versus* malignant). However, statistical analysis could not be conducted on Brenner type due to the small number of cases.

**Table 2:** Differences in stromal p16 expression between benign, borderline, and malignant ovarian lesions

Category	Total	p16 immunostaining score							*P* value
		0	1	2	3	4	6	9	
Benign	24	14 (58.3)	7 (29.2)	2 (8.3)	1 (4.2)	0 (0.0)	0 (0.0)	0 (0.0)	
Borderline	21	0 (00.0)	2 (9.5)	7 (33.3)	8 (38.1)	4 (19.1)	0 (0.0)	0 (0.0)	<0.001^a^
Malignant	46	0 (0.0)	0 (0.0)	0 (0.0)	0 (0.0)	3 (6.5)	21 (45.7)	22 (47.8)	<0.001^b^

a Benign versus borderline;

b Borderline versus malignant

**Table 3:** Differences in stromal p16 expression between benign, borderline, and malignant ovarian lesions for each histological type

Histological type	Category	Total	p16 immunostaining score							*P* value
			0	1	2	3	4	6	9	
Serous	Benign	6	4 (66.6)	1 (16.7)	1 (16.7)	0 (0.0)	0 (0.0)	0 (0.0)	0 (0.0)	
	Borderline	7	0 (0.0)	1 (14.2)	2 (28.6)	2 (28.6)	2 (28.6)	0 (0.0)	0 (0.0)	0.008^a^
	Malignant	16	0 (0.0)	0 (0.0)	0 (0.0)	0 (0.0)	2 (12.5)	10(62.5)	4 (25.0)	<0.001^b^
Mucinous	Benign	7	4 (57.1)	2 (28.6)	0 (0.0)	1 (14.3)	0 (0.0)	0 (0.0)	0 (0.0)	
	Borderline	8	0 (0.0)	1 (12.5)	2 (25.0)	3 (37.5)	2 (25.0)	0 (0.0)	0 (0.0)	0.008^a^
	Malignant	10	0 (0.0)	0 (0.0)	0 (0.0)	0 (0.0)	0 (0.0)	4 (40.0)	6 (60.0)	<0.001^b^
Brenner	Benign	3	3(100.0)	0 (0.0)	0 (0.0)	0 (0.0)	0 (0.0)	0 (0.0)	0 (0.0)	
	Borderline	1	0 (0.0)	0 (0.0)	1(100.0)	0 (0.0)	0 (0.0)	0 (0.0)	0 (0.0)	Not performed^c^
	Malignant	1	0 (0.0)	0 (0.0)	0 (0.0)	0 (0.0)	0 (0.0)	0 (0.0)	1(100.0)	Not performed^c^
Endometriosis-associated^d^	Benign	8	3 (37.5)	4 (50.0)	1 (12.5)	0 (0.0)	0 (0.0)	0 (0.0)	0 (0.0)	
	Borderline	5	0 (0.0)	0 (0.0)	2 (40.0)	3 (60.0)	0 (0.0)	0 (0.0)	0 (0.0)	0.004^a^
	Malignant	19	0 (0.0)	0 (0.0)	0 (0.0)	0 (0.0)	1 (5.3)	7 (36.8)	11(57.9)	<0.001^b^

a Benign versus borderline

b Borderline versus malignant

c Due to low sample size

d Endometriosis-associated type includes endometrioid, clear cell, and seromucinous types.

### Difference in stromal p16 expression between histological types

When ovarian neoplasms were classified by histological type without considering degree of malignancy, the p16 immunostaining scores varied from 0 to 9 points (Table 4). The mean p16 immunostaining scores of serous, mucinous, Brenner, and endometriosis-associated ovarian neoplasms were 4.3, 4.2, 2.2, and 5.4, respectively. No significant difference in stromal p16 expression was observed among the histological types. In addition, there was no statistically significant difference in stromal p16 expression between high-grade serous carcinomas and non-high-grade serous carcinomas (*P* = 0.267).

**Table 4:** Differences in stromal p16 expression between histological types

Histological type	Total	p16 immunostaining score							*P* value		
		0	1	2	3	4	6	9			
Serous	29	4 (13.8)	2 (6.9)	3 (10.3)	2 (6.9)	4 (13.8)	10(34.5)	4 (13.8)			
Mucinous	25	4 (16.0)	3 (12.0)	2 (8.0)	4 (16.0)	2 (8.0)	4 (16.0)	6 (24.0)	0.863^a^		
Brenner	5	3 (60.0)	0 (0.0)	1 (20.0)	0 (0.0)	0 (0.0)	0 (0.0)	1 (20.0)	0.150^a^	0.237^b^	
Endometriosis-associated^d^	32	3 (9.4)	4 (12.5)	3 (9.4)	3 (9.4)	1 (3.1)	7 (21.9)	11 (34.4)	0.337^a^	0.306^b^	0.089^c^

Versus

a serous

b mucinous

c Brenner type

d Endometriosis-associated type includes endometrioid, clear cell, and seromucinous types.

## DISCUSSION

A novel finding reported in this preliminary study is the gradual and significant increase in stromal p16 expression with increased degree of malignancy in benign, borderline, and malignant ovarian neoplasms. Consistent with this finding, comparing p16 expression within tumors of each histological type also revealed significant differences; in serous, mucinous, and endometriosis-associated neoplasms, stromal p16 expression in malignant and borderline lesions was significantly higher than that of borderline and benign lesions, respectively. In contrast, no significant difference was observed in stromal p16 expression among histological types. Our observation of significantly higher levels of stromal p16 expression in malignant ovarian lesions suggests that p16 may be involved in tumor cell growth and invasion in the tumor microenvironment through its overexpression in stromal cells. Some previous studies have reported p16 overexpression at the invasive tumor front of endometrial carcinoma, colorectal carcinoma, and basal cell carcinoma [17-20]. These results suggest that p16 may be involved in tumor invasion and angiogenesis and support the hypothesis that the p16 protein promotes invasiveness through interactions with other molecules related with tumor cell migration and invasion [1, 17-19, 21]. To confirm our preliminary results, it will be necessary to analyze stromal p16 expression using a larger number of ovarian carcinoma samples.

We found two previous studies reporting stromal p16 expression and its clinical implications in breast carcinoma [22] and endometrial neoplastic lesions [23]. For 80.0% (28/35) of endometrial polyp cases, p16 immunoreactivity with moderate or greater intensity was observed in fibrous stroma, and 1 (3.0%; 1/33) case of endometrial hyperplasia showed weak p16 expression; however, none of the endometrial carcinoma cases (0.0%; 0/23) showed stromal p16 expression. Moritani and colleagues [23] stated that stromal p16 expression was a characteristic finding of endometrial polyps and was useful in differentiating between endometrial hyperplasia and endometrial polyps. These results were inconsistent with our findings that stromal p16 expression was significantly higher in borderline and malignant lesions than in benign lesions. We attribute these differences to the following two reasons. First, two different sets of tissue samples (ovary and endometrium) were used, and stromal p16 expression patterns may be organ-specific. Second, p16 overexpression was reported to be observed in benign tumors such as benign nevus, neurofibroma, and schwannoma, which are known to be related to oncogene-induced cellular senescence [23]. Thus, p16 overexpression in benign lesions inhibited cellular proliferation, protecting cells from malignant transformation [1]. Significantly higher rates of stromal p16 overexpression in endometrial polyps can be explained by oncogene-induced cellular senescence. In contrast, in this study, malignant lesions showed a higher level of stromal p16 expression, which might be due to a positive feedback mechanism caused by RB protein deregulation. A study on p16 expression in the stroma of ductal carcinoma *in situ* of the breast [22] provided evidence that DCIS with high stromal p16 expression tended to show estrogen receptor negativity and high Ki-67 labeling indices. In addition, it was reported that high stromal p16 expression was a strong independent predictor of ductal carcinoma *in situ* recurrence with a higher hazard ratio than the established prognostic markers. These findings are in agreement with our data. p16 is an inhibitor of cell growth in response to various stress stimuli, such as DNA damage, oxidative stress, or hyperproliferative signals. Therefore, the p16 protein induces cellular senescence, such that stromal p16 overexpression is indicative of stromal cell senescence. Based on results of previous studies, [23-25], however, we postulated that senescent stroma can contribute to disease progression by secreting inflammatory mediators, cytokines, and enzymes such as proteases, providing a mechanism through which p16-positive stroma contributes to tumor progression and/or invasion.

In conclusion, we demonstrated that stromal p16 expression of malignant ovarian neoplasms was significantly higher than that of borderline ovarian neoplasms, which in turn was significantly higher than that of benign ovarian neoplasms. Stromal p16 expression was absent or weak in benign lesions, whereas the majority of malignant lesions exhibited diffuse and moderate-to-strong p16 immunoreactivity, suggesting that stromal p16 expression can be used as an adjunctive biomarker reflecting the development of ovarian carcinoma. Further studies are necessary to confirm our preliminary results.

## MATERIALS AND METHODS

### Tissue specimens

Ninety-one cases of ovarian lesions were retrieved from the surgical pathology files of Severance Hospital from March 2015 to May 2016. The pathological diagnoses are summarized in Table 1. Ovarian lesions were classified as benign, borderline, and malignant in 24, 21, and 46 cases, respectively. The age of patients ranged from 23 to 82 years (median, 50 years). Of the 24 cases of benign lesions, 11, 7, and 6 cases were diagnosed during bilateral salpingo-oophorectomy, unilateral salpingo-oophorectomy, and partial oophorectomy, respectively. Of the 21 cases of borderline lesions, 9, 8, and 4 cases were diagnosed during abdominal total hysterectomy with bilateral salpingo-oophorectomy, bilateral salpingo-oophorectomy, and unilateral salpingo-oophorectomy, respectively. Thirty-nine of the 46 malignant lesions cases were diagnosed in a primary debulking surgery (including laparoscopic or abdominal total hysterectomy, bilateral salpingo-oophorectomy, pelvic and/or para-aortic lymph node dissection, total omentectomy, and/or tumorectomy), and the remaining 7 cases were diagnosed in a unilateral salpingo-oophorectomy. This study did not include any cases where the histological differential diagnosis between benign and borderline lesions was ambiguous. This study was reviewed and approved by the Institutional Review Board at Severance Hospital, Yonsei University Health System, Seoul, Republic of Korea (2016-0931-001).

### Histopathological examination

The resection specimens were fixed in 10% neutral-buffered formalin and embedded in paraffin blocks. From each formalin-fixed, paraffin-embedded block, 4-μm sections were cut and stained with hematoxylin and eosin. Two independent pathologists examined all available hematoxylin and eosin-stained slides by routine light microscopy and chose the most representative formalin-fixed, paraffin-embedded block to perform immunohistochemical staining.

### Immunohistochemical staining

The formalin-fixed, paraffin-embedded sections were deparaffinized and rehydrated with a xylene and alcohol solution. Immunohistochemical staining was performed using a Ventana Benchmark XT automated staining system (Ventana Medical Systems, Tucson, AZ, USA), according to the manufacturer's instructions. Antigen retrieval was performed using Cell Conditioning Solution (CC1; Ventana Medical Systems). Sections were incubated with primary antibodies against p16 (pre-diluted, clone E6H4, Ventana Medical Systems). After chromogenic visualization, using UltraView Universal DAB Detection Kits (Ventana Medical Systems), slides were counterstained with hematoxylin. Appropriate positive and negative controls were concurrently stained to validate the staining method.

The percentage of p16-positive stromal cells and the staining intensity were assessed. A cut-off index was defined as the presence of 10% or more cells displaying nuclear p16 immunoreactivity, as previously described [23, 26]. The estimated percentages were categorized as follows: less than 10% (score 0), 10% to 24% (score 1), 25% to 50% (score 2), or 50% or more (score 3). The staining intensity was graded as follows: negative (score 0), weak (score 1), moderate (score 2), or strong (score 3). The subcellular location of p16-positive signals (nuclear or cytoplasmic) was also estimated. The final score was calculated as the multiplication of percentage and staining intensity, resulting in scores of 0, 1, 2, 3, 4, 6, and 9 [27].

### Statistical analysis

A linear-by-linear association test was performed to compare the status of stromal p16 expression between histological types and to determine whether stromal p16 expression was significantly different according to degree of malignancy. Statistical analyses were performed using the SPSS Software Package (version 18.0; IBM SPSS, Chicago, IL, USA). Statistical significance was set at *P* < 0.05.
